# Robust Optical Recognition of Cursive Pashto Script Using Scale, Rotation and Location Invariant Approach

**DOI:** 10.1371/journal.pone.0133648

**Published:** 2015-09-14

**Authors:** Riaz Ahmad, Saeeda Naz, Muhammad Zeshan Afzal, Sayed Hassan Amin, Thomas Breuel

**Affiliations:** 1 University of Technology, Kaiserslautern, Germany; 2 Shaheed Benazir Bhutto University, Sheringal, Pakistan; 3 Hazara University, Department of IT, Mansehra, Pakistan; 4 GGPGC No.1, Abbottabad, Pakistan; 5 Genie Technologies (Pvt) Ltd. Lahore, Pakistan; Xiamen University, CHINA

## Abstract

The presence of a large number of unique shapes called ligatures in cursive languages, along with variations due to scaling, orientation and location provides one of the most challenging pattern recognition problems. Recognition of the large number of ligatures is often a complicated task in oriental languages such as Pashto, Urdu, Persian and Arabic. Research on cursive script recognition often ignores the fact that scaling, orientation, location and font variations are common in printed cursive text. Therefore, these variations are not included in image databases and in experimental evaluations. This research uncovers challenges faced by Arabic cursive script recognition in a holistic framework by considering Pashto as a test case, because Pashto language has larger alphabet set than Arabic, Persian and Urdu. A database containing 8000 images of 1000 unique ligatures having scaling, orientation and location variations is introduced. In this article, a feature space based on scale invariant feature transform (SIFT) along with a segmentation framework has been proposed for overcoming the above mentioned challenges. The experimental results show a significantly improved performance of proposed scheme over traditional feature extraction techniques such as principal component analysis (PCA).

## 1 Introduction

The advent of information technology and large use of on-line digital text are increasing the demand of conversion of novels, newspaper, books and old manuscript into computer readable form. For this purpose, Optical character recognition (OCR) systems have been designed for non-cursive scripts like printed English, German, French and for cursive script based languages like Chinese, Korean, Arabic, Persian and Urdu etc.

Non-cursive scripts based languages are inherently easy for a machine to recognize, for example English, because the shape of individual character have very little variation for printed text. However the cursive scripts like Urdu, Arabic, and Pashto have complex word formation rules [1]. These complexities include shape variation of individual character, space insertion and space omission due to non-joiner letters [2], complexity in segmentation, base line detection etc. Another, in Arabic like scripts when characters join, they form intermediate shape instead of words. These intermediate shapes are called ligatures. Due to such complexities recognition of cursive languages are far away from the maturity level and most of the regional languages have not been explored due to complexity of cursive writing along with large number of ligatures or unique shapes. These ligatures are foundation units in cursive languages. In this research work we are dealing Pashto language as a test case.

Arabic and Pashto have similar nature e.g. their alphabet set is similar with some added characters in Pashto. Arabic writing is cursive in nature, in which characters are connected with cursive stroke. Moreover each character has two to four shapes depending on the position where it occurs. Words or ligatures are the foundation units of any cursive script language, Urdu has approximately 26,000 ligatures [3], similarly Pashto has a large number of ligatures.

OCR has different components i.e., optical scanning, location and segmentation, pre-processing, feature extraction, recognition and post processing. Since many errors may be introduced due to optical scanning, segmentation and pre-processing stages, therefore in this research we are focusing on feature extraction and recognition stages of the optical character recognition.

Research on OCR for cursive languages shows two main approaches for any OCR system [4]. (1) Analytical approaches and (2) holistic approaches. Analytical approaches are mainly based on grammatical rules and typographical principals for the respective script. However, these approaches need atomic segmentation for their efficient performance, while accurate segmentation is another complex issue. On the other hand holistic approaches are not based on typography rules and principals. Holistic approach are generic and can be easily applied to any text of any language. Our research is also based on holistic approach. In this approach we are trying to recognize ligatures in a holistic framework. More specifically our proposed approach is robust to scale, location and rotation variations. In general scale variations exist in all text documents, while rotation variations are also exist. The term optical character recognition abbreviated as OCR is relevant to recognition of individual symbols or alphabets in English and other languages, but for the recognition of cursive scripts using holistic/analytical approach the term optical recognition of cursive scripts abbreviated as ORCS is more relevant. In this research, we will be using ORCS to refer to recognition of the cursive script.

The extraction of features from a specific domain where the number of classes are in large number need extra care to extract rich set of features for discrimination. The features set should be compact, invariant to scaling, orientation, location/registration issues and reliable under noisy condition.

## 2 Related Work

The design of an OCR system is mainly dependent on the domain for which it is designed. The problem of cursive script recognition is similar to handwritten English. Work done on other cursive languages such as Arabic, Persian and Urdu OCR systems provide relevant literature for Pashto OCR system. In addition, we can also make use of work on Chinese, Hindi and graphical symbol recognition to discover useful techniques that can be applied for Pashto optical script recognition.

As discussed in the introduction section, we can categorize relevant research into analytical and holistic approaches [4]. The analytical approaches are based on the rules and on constraint of the specific text in a specific language. In these approaches the shape of each character is uniquely identified by its unique features like; hole, loop etc. Analytical approaches work very well in the text, where atomic segmentation is comparatively easy task. For example in printed English text, boundaries of each character can be found easily. In short, we need proper segmentation to achieve acceptable performance in analytical approaches. However, accurate segmentation of cursive scripts is challenging research issue. The differences between analytical and holistic approaches extend to the kind of data used for training and testing of the ORCS systems.

Hidden Markov model (HMM) and neural networks have been used frequently for recognition of cursive scripts [5]. Andrew Gillies et al [6] proposed Arabic text recognition system based on the analytical approach. The system has been tested on images of 138 pages of printed Arabic text at two different resolutions giving up to 93% correct recognition rate. The segmentation module of the system aims to extract atomic characters; however it may not always be successful resulting in recognition errors. Many of the ligatures are formed by joining characters in a non-standard way resulting in the failure of the segmentation stage. The segmented components are finally recognized using a neural network.

In ICDAR 2005, first competition for Arabic handwriting recognition was held in which five system trained on IFN/ENIT database [7]. In this competition highest recognition rates of around 80% to 90% were obtained, thus showing the need for further research in this area. In ICDAR 2007 [8], the result for Arabic handwriting recognition are further improved showing recognition rates greater than 90%. It is difficult to compare the characteristics of the systems on the basis of recognition results as details of many of the participating systems were kept secret.

In holistic approaches for cursive script recognition, we are not interested in atomic segmentation. The image having a word or ligature is holistically considered, in this way features are extracted and classification is made to assign a proper class to that ligature. In this technique we get rid of the segmentation problems i.e. checking of the boundaries and detection of turning point for each and every character. But we need to store all the valid ligatures or shapes for the given language for training purpose.

BBN Byblos OCR system is a holistic approach that does not require segmentation during training and testing. It is an HMM based approach that uses 14-state left to right HMM. The proposed approach is script independent and has been tested on multiple languages [9]. The authors provide character and word error rates which are quite low for synthetic data across five different fonts, however these rates scale up for real world data. They only model a few ligatures; the training and test data in their work are simply scanned images of Pashto documents.

Husain et al. [10] developed a multi-tier holistic approach for recognition of Noori Nastaliq script. The Nastaliq script are obtained through calligraphy and thus are more complex to render and to recognize as compared to Naskh scripts for cursive languages but are highly valued for their beauty. The proposed approach has been utilized to recognize ligatures from a pre-defined ligature set using features such as solidity, number of holes, axis ratio, eccentricity, moments, normalized segment length, curvature, ratio of bounding box width and height. The proposed system is trained and tested on synthetically created dataset. A feed forward back propagation neural network is used to perform the final recognition task giving high recognition rates.

It must be pointed out here in addition to font variations; printed text usually contains scaling and rotation variations. These kinds of variations are challenging to overcome, so far none of the above approaches have incorporated these kinds of variations into the training dataset, and we have no results that can show effectiveness of various methods against these challenges. One of the main objectives of the proposed approach is to overcome scaling, rotation and location variations in the printed text.

## 3 Pashto Image Database

For any recognition system it is very important that it must have a rich data set. In case of OCR systems, dataset must have all the valid data regarding specific language. Pashto is an ancient language having almost 44 alphabets with lot of variation while connected to other characters. Another key point is the ligature base approach, which requires basic character set as well as the ligatures of the Pashto. So acquiring a dataset with rich image base is essential for Pashto OCR system.

Databases and competitions play very important role in development of practical recognition systems [11]. Datasets can be created in two ways i.e. synthetic image databases and real image databases. To collect handwriting samples, special forms have been used, which are then scanned. Similarly CEDAR database provides real world data containing bank cheques and postal addresses. Synthetic image datasets require comparatively less effort because these avoid data collection, scanning and are relatively free from various artifacts. These are also well suited for training and testing because of their organization. However, a system trained on synthetic data needs to be adapted to give good performance for the real world data.

A key issue when selecting a rich dataset is the instances of same classes with its appropriate variations. Literature review shows that, for certain approaches very limited datasets were used, and it was also observed that there is no such dataset which provide enough support for testing several variations like position, scaling and orientation.

In Multi-Tier Holistic approach for recognition of Urdu ligatures, scaling and rotation variations are not included in image dataset. Image dataset used in this research is very limited having images of just 200 ligatures [10].

Pal and Sarkar [12] proposed an analytic approach that has been trained on 3050 characters. The system recognizes basic characters and numerals with 97%, however it suffers from many segmentation errors. Furthermore, the database is not well suited for holistic approaches.

## 4 Background and Characteristics of Pashto Script

Pashto is an ancient language that is spoken by over 50 million people in all over the world [14]. However, it is mostly spoken in Afghanistan and Pakistan. In Afghanistan, it is the national language; whereas Pakhtuns also live in large numbers in Baluchistan and Khyber Pakhtunkhwa province of Pakistan.

**Fig 1:**
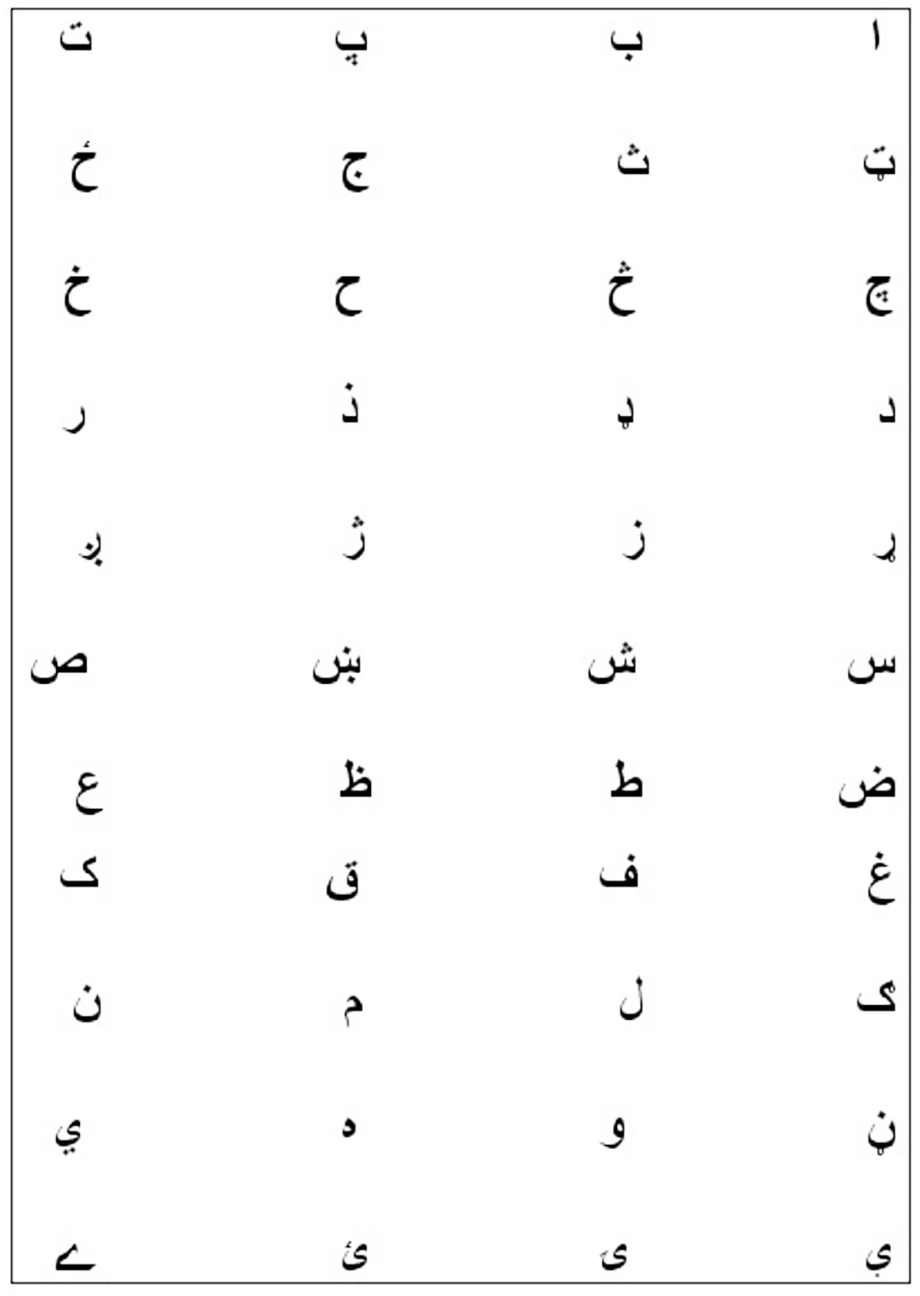
Pashto Characters. Pashto has 44 basic Alphabets [13].

**Fig 2:**
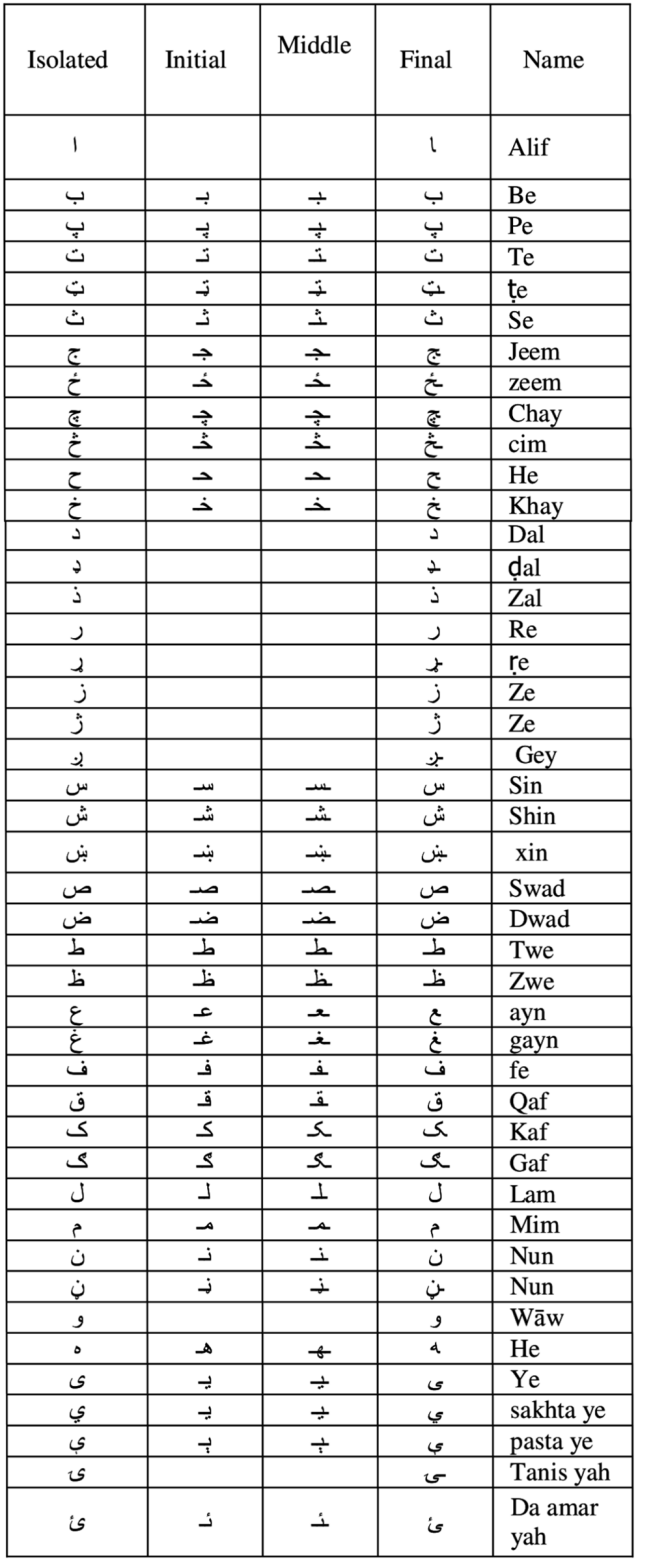
Shape variation of characters within Pashto character set [13].

It is a cursive language that is written in Arabic style. Pashto character set contains 44 alphabets (see Fig 1). Interestingly, alphabets of Arabic, and Persian languages are the subset of Pashto character set. Being a cursive language alphabets change shape when forming a ligature. Thus each alphabet may have up to four shapes depending on its position in the ligature. These four shapes can be describe as (1). Shape of the isolated character, (2). Shape of the initial character, (3). Shape of the middle character, (4). Shape of the last or final character. Shape of the isolated characters are shown in Fig 1, whereas shapes of the Pashto characters that vary according to their position within ligature are shown in Fig 2.

Pashto is mostly written in Naskh style which is rule based, and thus easier to render and typset. The fonts for Pashto include Abdaali, Kandhar, Waziristan and Karor.

### 4.1 Problems in Recognition of Pashto Cursive Script

Both analytical and holistic approaches have certain advantages and disadvantages. In analytical approach segmentation is a key to accurate recognition; the recognition stage is thus simplified. Whereas in holistic approach, accurate segmentation is obtained as a by-product of the recognition, but the recognition stage is challenging. Here, we outline key challenges that are common to both the approaches but are more relevant to the holistic approach being used in this research.

#### 4.1.1 Location Sensitivity

**Fig 3:**
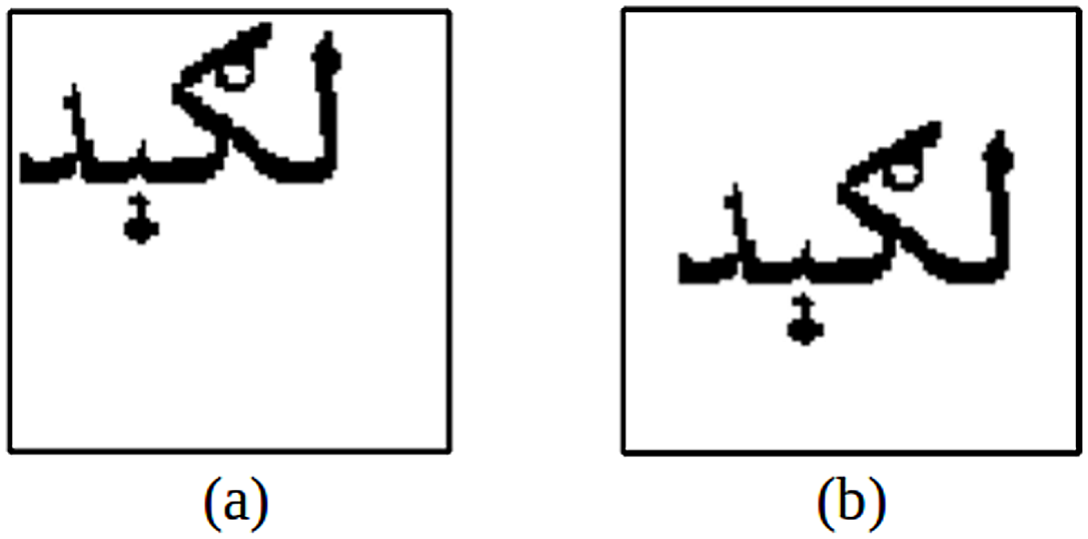
Location variation; Ligature in (a) is in test set, while (b) shows the same ligature in training set.

The location sensitivity is related to the position of a ligature in the given image. For example in a database, there is an image of a ligature “Lageed”, and the position of this ligature is at the centre of the image, but the test image have the same ligature but on a position at corner, then PCA gives us very poor result. This problem is also known as registration variation and is shown in the Fig 3. This problem is mainly encountered in PCA based methods, because PCA is sensitive to image registration. Though, the PCA and it’s variant like Ranking Sensitive PCA along with Ranking Sensitive Vocabulary Boasting are used in searching landmarks in mobiles [15].

#### 4.1.2 Scaling Sensitivity

**Fig 4:**
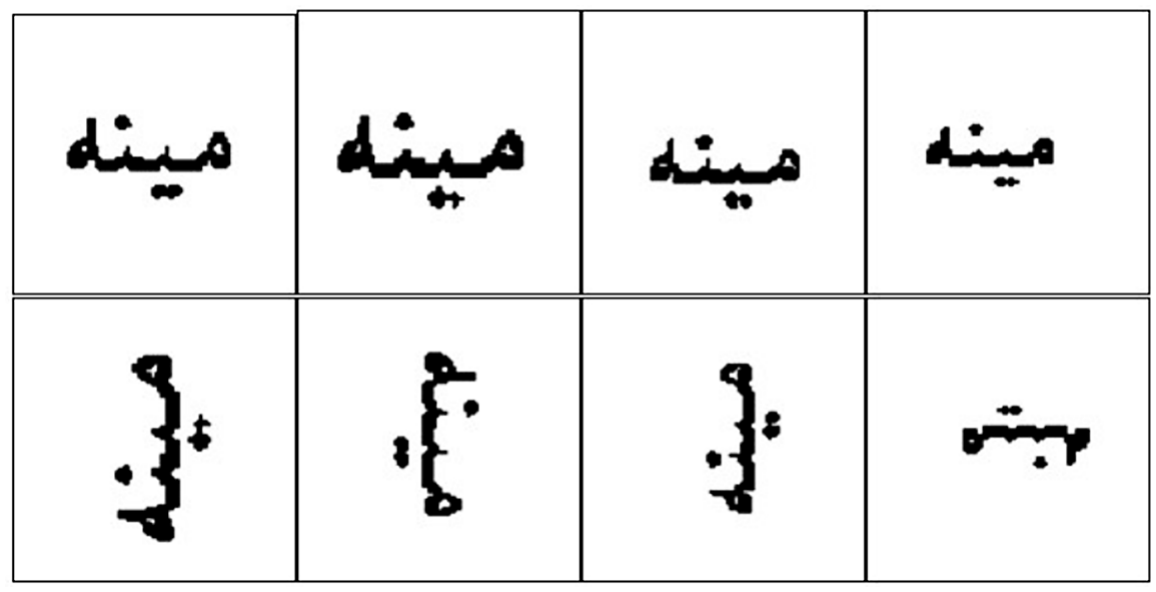
Scale and orientation variations in Pashto image dataset.

Font size variation is one of the important features of typical text images, because we can see a same ligature having different sizes in headers and normal text. This is a key requirement for recognition of the real world data. In many cases the reason for poor recognition results for classification methods such as PCA is scaling variation as shown in the Fig 4.

#### 4.1.3 Orientation Sensitivity

Rotation or orientation variation is a major challenge, because as seen in real world text images, the text are in a rotated form. In some cases the documents having text may be rotated. Feature extraction techniques such as PCA are highly sensitive to rotation; Fig 4 also shows the orientation problem.

### 4.2 Image Database of Pashto Ligatures

Image databases for optical recognition of cursive languages differ a lot in terms of font styles, image resolution and quality, content (page, single ligature) and acquisition method. If the data has been acquired from real world data such as a printed page, book or newspaper using scanner then it is categorized as real world data.

Computer Science department of NUCES FAST Peshawar campus has been working on Pashto OCR system since 2006. This research has resulted in creation of Pashto image dataset, which has 4000 images of 1000 unique ligatures along with scaling variation for font size 12, font size 14, font size 16 and font size 18. The font being used for type setting the ligatures is ‘Karor’. The image resolution is 100 by 100. The proposed image dataset has been created synthetically using computer generated images [16].

We are also using this dataset for training and testing our proposed approach. To address the orientation problem, there is need for images of Pashto ligatures in rotated form, such that robustness of the recognition system against orientation can be verified.

To address orientation problem we have added rotated images of ligatures. After adding these rotated ligatures now our dataset contains 8000 ligatures with 4 different scaling and four different orientation variations. The database also contains location variations for each ligatures [13]. The database is publicly available, and can be obtained from the authors by sending an email request to the authors.

The synthetically created dataset being used in this research is suitable for research using not only holistic approach, but it can also be very useful for training and evaluation in analytical approach. The success of the segmentation stage can be easily verified by testing it on large number of ligatures available in this database.

## 5 Outcomes of Robust Recognition System for Pashto Script

In this section, we describe feature space and matching technique being used in this research. First, a simple recognition approach is introduced, and its results are compared against principal component analysis. Then limitation of simple SIFT-based ORCS are explained and component based Pashto recognition scheme based on SIFT feature is presented. The experiments section describes the effect of feature space on recognition rates given scaling, orientation registration differences among training and testing images. Various factors effecting recognition rates are discussed and improvements are made in proposed scheme where possible.

### 5.1 Invariant Features for Pashto

While discussing object recognition system, focus must be on the detection of a set of features that play vital role in classification as well as recognition. This research is focused on scaling, location and orientation sensitivity of feature space. So we need an efficient algorithm that can extract such features point from a text image, which are invariant to scaling, location and orientation.

David Lowe in 1999 introduced an algorithm called SIFT (Scale Invariant Features Transform) [17]. SIFT applications include object recognition, robotic mapping and navigation, image stitching, 3D modelling, gesture recognition and video tracking. The core advantage of SIFT is the extraction of such features which are invariant to scaling, rotation and registration/location of ligatures.

It is very important to know the practical work and results of SIFT descriptor in the field of image recognition. Particularly, we will need the results of SIFT regarding scaling and orientation. In [18], David Lowe applies SIFT technique to object recognition problem where scale, rotation and location are allowed to change; and we may also be dealing with partial occlusions and illumination variations. The experimentation shows that despite differences of scale, rotation, and illumination high percentage of matching points are detected between the images. Similarly, Zhang et al [19] apply a modified SIFT approach called character-SIFT for the recognition of off-line Chinese character. The character SIFT approach obtains a recognition rate of 97.86% on HCL2000 database of Chinese characters containing 3755 simplified Chinese characters. Recent research shows that SIFT has been using in providing BOF “bag-of-features” for Mobile Visual Location Recognition (MVLR) [20] [21] [22] [23]. Similarly, to speed up the features based matching, the use of algorithm for Approximation of Nearest Neighbour (ANN) based on Residual Vector Quantization are proposed [24]. These features descriptors can be represented by visual-codebooks. The conversion of features descriptor into discrete visual codebooks are obtained by quantization. However, the issues related to the sparse data from large scale features descriptors are addressed by compression technique mentioned in [25]. In addition, the indexing and distribution of these gigantic visual vocabulary across different servers can be managed and parallelized by approach mentioned by Rongrong Ji et al. [26].

This research explores the use of SIFT descriptor for recognition of Pashto ligatures. SIFT descriptors are widely used in computer vision community and have been applied to handwritten character recognition problem, but so far have not been applied to recognition of cursive scripts in a holistic framework.

SIFT is comprised of four stages, (1) Scale-space extrema detection, (2) Key-point localization, (3) Orientation assignments and (4) Key-point descriptor. We have used the SIFT technique on our dataset and found it, as an appropriate candidate approach for our problem.

**Fig 5:**
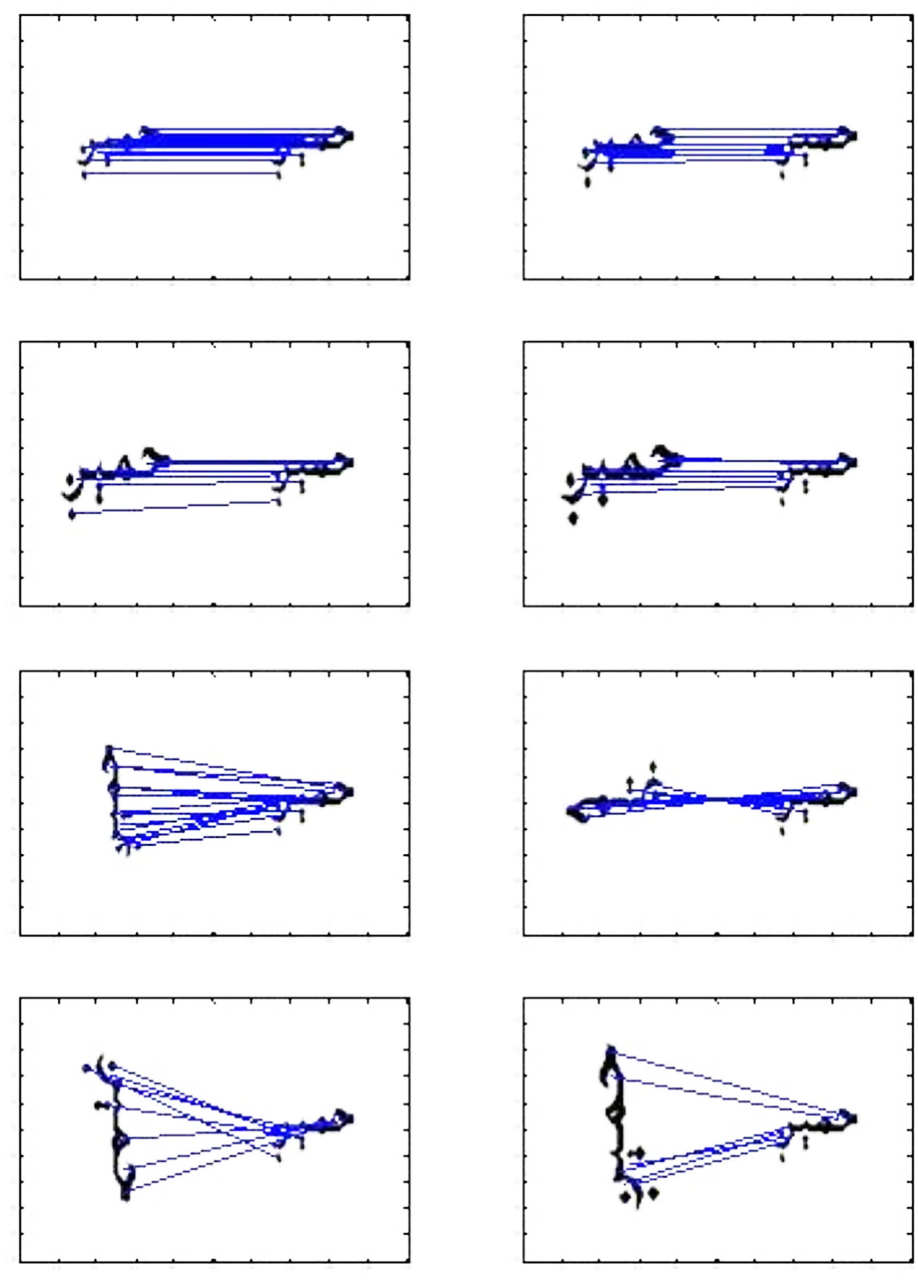
Illustration of Ligature matching with all available ligatures against SIFT-S1 descriptor.

In this research, SIFT descriptor is calculated and stored for each of the training images during the training phase. The SIFT descriptor will be calculated for the test image during test phase. Training and test images will have different number of key-points. The target image is matched by finding maximum numbers of key-points; which were found matched with the key-points of the candidate test image. Fig 3 shows a ligature being matched with ligatures having different scale, location and orientation. In this case SIFT-S1 has been used for training. The Fig 5 shows lines emanating from key-points in test image and connecting to matching key-points in the corresponding image of training dataset having different size and rotation.

### 5.2 Experimental Investigation for Simple SIFT Approach

The main objective of the experimentation is to find out effectiveness of SIFT for overcoming scaling and orientation challenges. To explain experimental setup, we are introducing some conventions that are used in confusion matrices, figures as well as in text. S1, S2, S3 and S4 refer to images created using font size 12, 14, 16 and 18 respectively. S1R, S2R, S3R and S4R refer to images of rotated ligatures (±90 degrees) created using font size 12, 14, 16 and 18 respectively, see Figs 4 and 5.

We have used MATLAB to generate SIFT descriptor for all font sizes. SIFT-S1, SIFT-S2, SIFT-S3 and SIFT-S4 are the SIFT descriptors of their respective sizes for training image database. In this way, all these SIFT descriptors were generated and stored permanently. Our classification approach is based on finding maximum numbers of key-points; which were found to be matched with the key-points of the test image.

**Fig 6:**
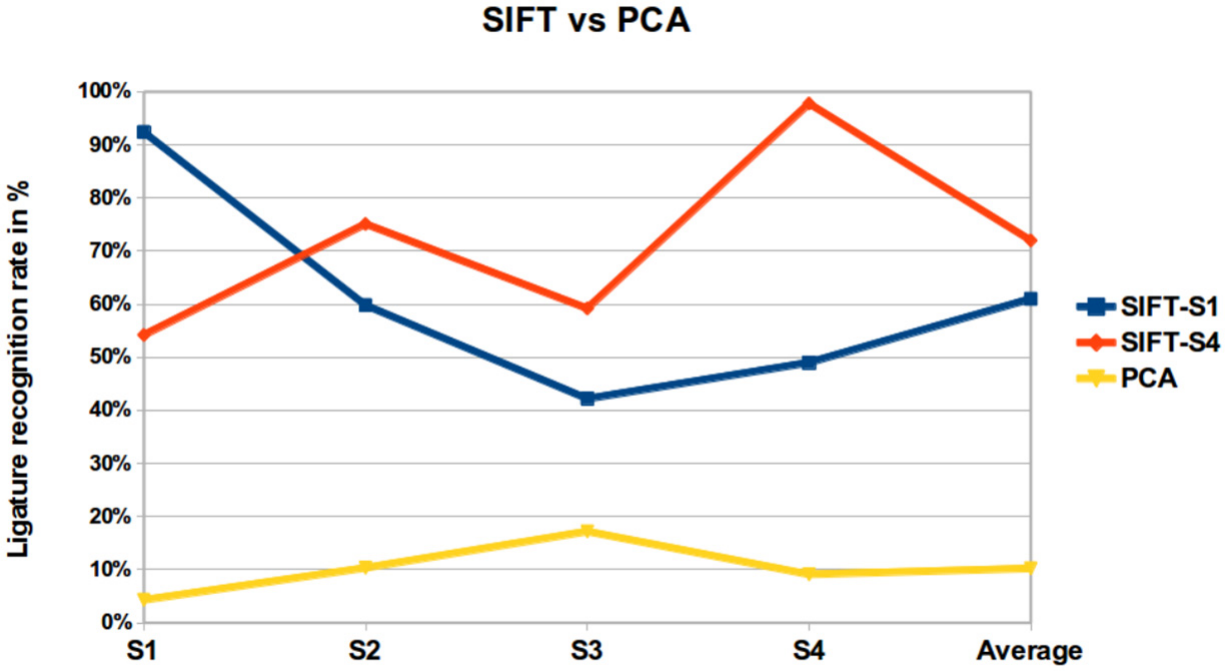
In case of PCA, the recognition rate has been obtained using font size S2 and S3, whereas in case of SIFT approach recognition rate has been obtained using font size S1 and S4.

The experimental results for SIFT approach are shown in the Tables 1 and 2. These results are compared against PCA in Fig 6. For PCA, training has been done using two font sizes, whereas in case of SIFT training has been done using just one font size. The experimental results therefore clearly show that SIFTS performs much better when dealing with typically occurring variations of various kinds. In subsequent sections, we have investigated scaling/location and orientation/location issues experimentally.

**Table 1:** Confusion matrix.

	S1	S2	S3	S4	Average
*SIFT* − *S*1	92.40%	59.80%	42.20%	49.0%	61.0%
*SIFT* − *S*2	59.40%	96.90%	66.10%	74.40%	74.0%
*SIFT* − *S*3	44.20%	57.00%	95.70%	63.60%	65.0%
*SIFT* − *S*4	54.20%	75.10%	59.20%	97.78%	72.0%

Ligatures having sizes S1, S2, S3 and S4 are shown along y-axis, as a test data, and along x-axis as training data. The correct classification rate (%) is shown at intersections of training and test data [16].

**Table 2:** Confusion matrix.

	S1R	S2R	S3R	S4R	Average
*SIFT* − *S*1	92.70%	59.10%	41.50%	48.0%	60.0%
*SIFT* − *S*2	57.30%	96.90%	66.80%	72.50%	73.0%
*SIFT* − *S*3	42.10%	59.50%	95.20%	64.10%	65.0%
*SIFT* − *S*4	51.70%	74.80%	59.70%	97.50%	71.0%

Sizes S1R, S2R, S3R and S4R with rotated ligatures are shown along x-axis, as a test data. The correct classification rate (%) is shown along x-axis for each distinct SIFT descriptor [16].

#### 5.2.1 Experimental Evaluation of Scaling/Registration Invariance

In this experiment, the scaling/registration issue is investigated. All SIFT descriptors of the selected font size are compared against images obtained from other font sizes. The confusion matrix in Table 1 compares classification rate in (%) with font size (scaling) variation. The registration variation is implicit with scaling variation. We can see that the SIFT descriptor obtained from S2 (i.e. font size 14) gives comparatively better recognition results. The diagonal terms indicate acceptable performance against similar data; however off-diagonal terms vary significantly depending on the training data with results far better for font size 2.

#### 5.2.2 Experimental Evaluation of Orientation/Registration Invariance

In this experiment, the orientation/registration issue is investigated. Once again registration variation occurs because of rotation variations. In this experiment, the available SIFT descriptors (SIFT-S1, SIFT-S2, SIFT- S3 and SIFT-S4) are compared against SIFT descriptors for rotated images of S1R, S2R, S3R and S4R sizes. The confusion matrix in Table 2 shows the comparison of classification rate (%) against orientation variations at different font sizes. The off-diagonal terms in the Table 2 shows classification rate varying between 41% to 74% which is comparatively better than PCA, but far from satisfactory. The diagonal terms indicate recognition rate in the range of 92% to 97% which is encouraging but further improvement is desirable. As discussed earlier, we do not have similar work in literature so there is need to address these issues to make progress on recognition of cursive scripts.

### 5.3 Limitations of Simple SIFT Approach

Experimentation have found some limitation regarding our dataset and SIFT technique. In our dataset the images/ligatures having font size 14 has good quality compare to other sizes. And the images/ ligatures having font size 16 have low quality. Therefore we can see the performance of SIFT-S2 is much better than other SIFT descriptors.

On the other hand SIFT is very sensitive to image resolution. For example, if an image having 80x80 resolutions can produce “k” number of SIFT key-points. But for the same shapes, if the resolution is set to 100x100 pixels, then SIFT can generate “n” number of key-points; where “n” will be always greater than “k”. In general constant number of key points are required for recognizing a specific shape, but SIFT fails to do so. Because SIFT gives different number of key- point in different sizes as well as in different resolutions.

Another abstract phenomenon is faced about the question that what size from the available dataset should be considered for training data. The smallest size gives us minimum key-points, and there is probability of missing some important key-points that can be obtained from large scales. On the other hand the selection of large scale size as trained data increases the probability of the competition for the candidate target image.

### 5.4 SIFT Component based Clustering Approach for Pashto Script Recognition

**Fig 7:**
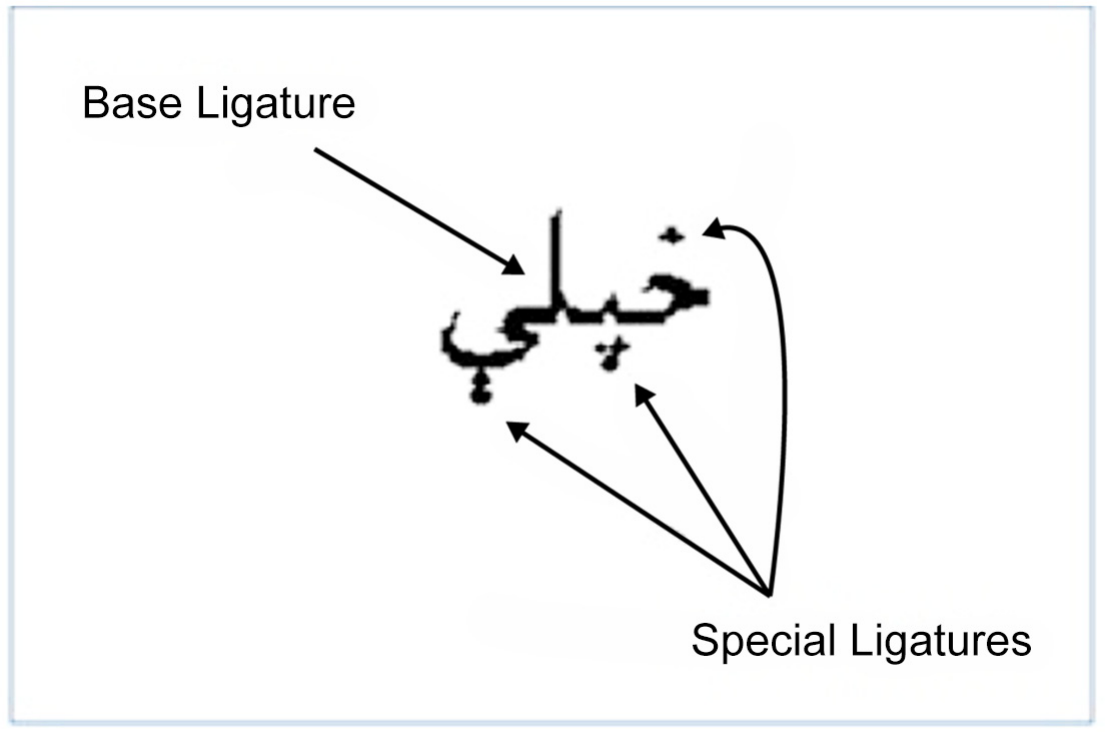
Base and Special Ligatures.

As shown in earlier research and limitations of SIFT based approach, components of ligature known as base and special ligature can be instrumental in improving the performance of the recognition system. In this research, we refer to base ligature and special ligature as component and obtain number of components for each ligature. Total “number” of these components and the “position” of special ligatures can play vital role to distinguish the similarities. There is always only one base ligature, but some ligatures may only differ by number and type of special ligatures. Fig 7 illustrates the combination of these components.

In our dataset simple ligatures have just 1 component i.e. only one base ligature and no special ligatures and similarly a set of ligatures having maximum number of components are up to 5 components. Thus 1 base ligature and 3 special ligatures are shown in Fig 7 is an example of components in a ligature. Thus using this heuristics we can divide our 1000 unique ligatures into 5 clusters. The enhanced approach is referred to as SIFT-Component Based Clustering (SCBC). So our enhanced version of proposed approach is mainly based on the exploitation of components in ligatures. In this approach first we obtain a component descriptor for each font size. Then we have stored these component-descriptors permanently. These component-descriptors have a number (from 1 to 5) that represent number of components in a specific ligature.

Classification is obtained by getting number of components of an input image. Then search the component descriptor to obtain number of ligatures in an array having same number of component as of input image. Then this array is searched for maximum score of SIFT, where successful hit will yield correct classification. SCBC scheme not only improve the recognition rate but also reduce time complexity, due to limited search space for matching.

#### 5.4.1 Experimental Evaluation of Scaling/Registration Invariance

This experiment evaluates the recognition performance of SCBC approach against scaling/registration variation. As shown in the Table 3, there is significant improvement in the recognition rates. The diagonal shows recognition rates in the range of 95% to 99%, whereas off-diagonal terms which are more significant show recognition rates in the range of 54% to 81%. These results confirm the ability of the SCBC approach to overcome scaling differences to recognize text in different font sizes.

**Table 3:** Confusion matrix.

Plain Text	S1	S2	S3	S4	Average
*SIFT* − *S*1 − *SCBC*	95.90%	68.90%	54.90%	59.8%	69.87%
*SIFT* − *S*2 − *SCBC*	71.20%	99.70%	77.90%	77.3%	81.52%
*SIFT* − *S*3 − *SCBC*	60.50%	76.10%	99.40%	74.90%	77.72%
*SIFT* − *S*4 − *SCBC*	61.10%	81.20%	72.30%	99.80%	78.60%

Sizes S1, S2, S3 and S4 with non-rotated ligatures are shown along y-axis, as a test data. The correct classification rate (%) is shown along x-axis for each distinct SCBC descriptor.

#### 5.4.2 Experimental Evaluation of Rotation/Registration Invariance

In this experiment, invariance against rotation/registration variance is evaluated by training using S1, S2, S3 and S4 and testing against arbitrary rotated ligatures S1R, S2R, S3R, and S4R. The results of the experimentation are shown in the Table 4.

**Table 4:** Confusion matrix.

Rotated Text	S1R	S2R	S3R	S4R	Average
*SIFT* − *S*1 − *SCBC*	98.10%	69.00%	54.90%	58.60%	70.15%
*SIFT* − *S*2 − *SCBC*	70.80%	99.50%	77.30%	76.60%	81.05%
*SIFT* − *S*3 − *SCBC*	58.70%	79.10%	99.40%	76.20%	78.35%
*SIFT* − *S*4 − *SCBC*	60.90%	80.50%	72.40%	99.80%	78.40%

Sizes S1, S2, S3 and S4 with non- rotated ligatures are shown along y-axis, as a test data. The correct classification rate (%) is shown at intersection of two axis for each distinct SCBC descriptor.

The diagonal shows high recognition rates that are mostly close 99%, whereas non-diagonal terms also show comparatively better results in range of 54% to 81%. The improvement obtained in the recognition rates as result of component based approach confirms that we can obtain high recognition rates irrespective of rotation variations using SIFT-based feature extraction and matching.

Furthermore, new methods like deep learning, which are based on Long Short Term Memory (LSTM) Recurrent Neural networks are effectively using for sequence learning tasks [27] [28]. Similarly, for Arabic and Urdu like scripts, LSTM based approaches have shown good accuracy rates ranging from 89% to 94% [29] [30] [31]. However, none of the works has been found, which is focusing on the invariance recognition of cursive scripts with respect to scale, rotation and registration variations. Therefore, technically these results are not comparable with the results of our proposed technique.

## 6 Conclusion

A wide variety of techniques have been proposed for recognition of cursive scripts such as Arabic, some of the work has been extended to Urdu, Persian and Pashto. As seen in literature review, the major research challenges in this area are font invariance, scale invariance, rotation invariance and location invariance. So far, very little progress has been achieved towards addressing these issues. In this paper, we present a holistic approach for scale, rotation and location invariant recognition of Pashto script. An image database of Pashto having font sizes 12, 14, 16 and 18 in Pashto Karor font has been used in this research. Additionally, rotated images of each ligature have been added to the database for rotation invariance. Selection of suitable feature space, that can provide a rich feature space having invariance for rotation, scaling and location for large number of ligatures is a major challenge. In this research, we propose scale invariant feature transform (SIFT) for recognition of Pashto ligatures. Two different approaches for recognition have been proposed. In each case, training is done on a single font size. In many cases, simple recognition approach gives relative poor recognition rates due to occurrence of multiple special ligatures. To overcome, these challenges a component based clustering approach is proposed. The proposed approach gives 81% accurate recognition rate providing high invariance for scaling, rotation and location.

A key advantage of the proposed approach is that it can be easily extended to other cursive scripts since it does not require knowledge of the rules for forming words. Only requirement for extending the proposed approach to other languages is availability of suitable image database.

In future, we hope to make improvements to the methodology for higher recognition rates by exploring the new emerging technique like deep learning. However, the implementation of deep learning approach requires large number of data. Therefore, we also need to extend Pashto dataset, such that to exploit the potentials of deep learning approach in an efficient way.
